# Ultrasound guided platelet rich plasma injections for post-traumatic greater occipital neuralgia following concussion: a pilot randomized controlled trial

**DOI:** 10.3389/fneur.2024.1400057

**Published:** 2024-06-07

**Authors:** Jacqueline E. Stone, Christina Campbell, Jason B. Tabor, Stephan Bonfield, Matthew Machan, Rodney Li Pi Shan, Chantel T. Debert

**Affiliations:** ^1^Department of Clinical Neurosciences, Cumming School of Medicine, University of Calgary, Calgary, AB, Canada; ^2^Hotchkiss Brain Institute, University of Calgary, Calgary, AB, Canada; ^3^Sport Injury Prevention Research Centre, Faculty of Kinesiology, University of Calgary, Calgary, AB, Canada

**Keywords:** greater occipital neuralgia, post-traumatic headache, concussion, traumatic brain injury, platelet rich plasma, corticosteroids

## Abstract

**Background:**

Treatment for post-traumatic greater occipital neuralgia (GON) includes serial injections of steroid/anesthetic. While these injections can alleviate pain, effects can be transient, frequently lasting only 1 month. As a potential alternative, platelet-rich plasma (PRP) injections are an emerging biological treatment with beneficial effects in peripheral nerve disorders. We investigated the feasibility, safety, and effectiveness of a single PRP injection for post-traumatic GON in comparison to saline or steroid/anesthetic injection.

**Methods:**

In this pilot randomized, double-blinded, placebo-controlled trial, 32 adults with post-traumatic GON were allocated 1:1:1 to receive a single ultrasound-guided injection of (1) autologous PRP (2) steroid/anesthetic or (3) normal saline. Our primary outcome was feasibility (recruitment, attendance, retention) and safety (adverse events). Exploratory measures included headache intensity and frequency (daily headache diaries) and additional questionnaires (headache impact, and quality of life) assessed at pre-injection, 1 week, 1 month, and 3 months post-injection.

**Results:**

We screened 67 individuals, 55% were eligible and 95% of those participated. Over 80% of daily headache diaries were completed with 91% of participants completing the 3-month outcome questionnaires. No serious adverse events were reported. There were no significant differences between groups for headache intensity or frequency. Headache impact on function test-6 scores improved at 3 month in the PRP (*β* = −9.7, 95% CI [−15.6, −3.74], *p* = 0.002) and saline (*β* = −6.7 [−12.7, −0.57], *p* = 0.033) groups but not steroid/anesthetic group (*p* = 0.135).

**Conclusion:**

PRP is a feasible and safe method for treating post-traumatic GON with comparable results to saline and steroid/anaesthetic. Further trials with larger sample sizes are required.

**Clinical trial registration:**https://clinicaltrials.gov/, identifier NCT04051203.

## 1 Introduction

Each year, over 40 million people suffer a mild traumatic brain injury (mTBI) worldwide (1). Approximately 30% of these individuals develop post-concussive symptoms lasting over 1 month, meeting International Statistical Classification of Disease and Related Health Problems, 10th revision (ICD-10) criteria for post-concussive syndrome (2, 3). The sequalae of persistent post-concussion symptoms (PPCS) includes a variety of somatic, psychological, cognitive, and neurological symptoms, with headache being one of the most common and debilitating (3–5).

Headache attributed to trauma or injury to the head and/or neck can greatly impact recovery, function, and quality of life. In the first year following concussion, over 70% of individuals experience headaches that may persist for decades following injury (4, 5). The pathophysiology of these headaches remains poorly understood, and treatment often consists of trial/error and combining therapies utilized for primary headache types (5, 6).

Occipital neuralgia (ON) is a subtype of headache attributed to trauma or injury to the head and/or neck (7–11). Diagnosed using the International Classification of Headache Disorders 3rd Edition (ICHD-3) (6), it is postulated that the pathogeny of ON results from damage or irritation along the occipital nerve (12, 13). Patients with ON report unilateral or bilateral paroxysmal, shooting, or stabbing pain. These symptoms can present in the distribution(s) of the lesser, and/or third occipital nerve, but most commonly in the greater occipital nerves (6, 12).

Serial perineural injections with steroids and/or anesthetic are standard treatment for Greater ON (GON) (14), and a beneficial response is required to confirm diagnosis (6, 12). Although effective, the average duration of pain relief is 1 month, often necessitating multiple injections (12, 13). These injections can become less effective over time and repeated exposure to steroid can have detrimental local and systemic effects (15). Consequently, many other treatments have been investigated, including oral medications, botulinum toxin injection, pulsed radiofrequency ablation, occipital nerve stimulation, and surgical decompression, all with variable degrees of invasiveness and success (12).

Given the lack of effective long-term treatment strategies for post-traumatic GON, we sought to evaluate an emerging biologic therapy: platelet rich plasma (PRP) (16). PRP is prepared from autologous whole blood and contains supraphysiologic concentrations of platelets, plasma, and associated growth factors. The use of PRP as an interventional treatment for many musculoskeletal disorders is well documented (17–21). Recently, PRP has shown potential as a treatment for peripheral nerve disorders (22, 23) and trials have demonstrated promising results for PRP’s ability to reduce pain in peripheral neuralgia (24–27). The safety profile, anti-inflammatory and regenerative properties of PRP make it an attractive therapeutic modality for post-traumatic GON with the potential for longer duration of effect over conventional steroid treatment (16, 18–20).

To date, PRP has not been investigated as a treatment for post-traumatic GON. Therefore, the primary objective of this pilot trial was to evaluate the feasibility of our study protocol and the safety of a single perineural PRP injection in treating post-traumatic GON following concussion. Feasibility and safety were determined through study recruitment, adherence, retention, acceptability, and reporting of adverse events. Our secondary objective was to compare the effectiveness of PRP versus saline and steroid/anesthetic injections on headache burden up to 3 months post-intervention.

Based on previous literature regarding the safety profile of PRP, we hypothesized that the study design would be feasible with minimal adverse events related to the study injection. We also hypothesized that PRP would be as effective as saline and steroid/anesthetic injections but may provide longer benefit given findings in other studies (18–20).

## 2 Methods

### 2.1 Overview

This prospective, randomized, controlled, double-blinded pilot trial was completed between June 2019 and December 2022, in Calgary Alberta. The trial was registered on ClinicalTrials.gov (NCT04051203) and approved by the University of Calgary Conjoint Health Research Ethics Board (REB 18-1,369). The methods for this study have been published elsewhere by the authors as a protocol paper (16).

### 2.2 Participants and setting

Participants were recruited from the Calgary Brain Injury Program, Calgary Chronic Pain Centre, Alberta Neurological Centre, through community neurologists and advertising on the University of Calgary Brain Neurorehabilitation Laboratory website.

Eligible participants were at least 18 years of age, met ICHD-3 criteria for headache attributed to trauma or injury to the head and/or neck and post-traumatic GON (6), as established by a physiatrist and/or neurologist, and had a previous beneficial diagnostic anesthetic injection to the greater occipital nerve. Participants were enrolled at least 3 months after their last GON injection and had an average pre-treatment daily headache intensity of ≥4/10 on Numeric Pain Rating Scale (NPRS) scale (on the days when headaches were present) and headache frequency of ≥10 days/month, chosen to reflect post-traumatic GON. All patients had a clinical diagnosis of mTBI, meeting the American Congress of Rehabilitation Medicine guidelines (28).

Exclusion criteria included: an inability to provide informed consent, a history of surgery in the occipital region, an unstable psychiatric or medical condition, uncontrolled rheumatologic or inflammatory disorders, widespread neurologic disorders (e.g., Multiple sclerosis), fibromyalgia/chronic fatigue syndrome, coagulopathy, immunosuppression, active cancer, herpes zoster infection within last 6 months, pregnancy, or if currently breastfeeding.

### 2.3 Clinical assessments

Demographic information and written/electronic consent were collected 2 weeks prior to study injection in person or by phone call (minimizing hospital visits during the COVID-19 pandemic). This included age, sex, height and weight, education, past-medical history, previous treatments, TBI history (date and mechanism of injury, previous head injuries, immediate and current symptoms) and headache history (frequency, intensity, headache type, medications, headache triggers and associated symptoms). Pre-injection Headache Impact Test-6 (HIT-6) (29) and Quality of Life After Brain Injury questionnaire (QOLIBRI) (30) were also administered at this time. Following initial assessment, 2 weeks of pre-injection daily headache diaries recording frequency and NPRS scores were completed. Headache diaries were completed daily for 3 months post-injection and HIT-6 and QOLIBRI were repeated at 1 week, 1 month, and 3 months post-injection. All questionnaires and any electronic consent forms were collected through online survey invitations using REDCap v.7 electronic data capture tools hosted at the University of Calgary (31, 32).

### 2.4 Feasibility and safety

Feasibility of the study protocol was determined by recruitment (ability to recruit more than 30% of those screened), appointment attendance (greater than 70% of those eligible consent and receive study injection), and retention (at least 70% of daily headache diaries completed and at least 70% of participants completing the 3 month HIT-6 and QOLIBRI). We determined the safety of the PRP injections by monitoring adverse events documented by participants in the additional comments section of daily headache diaries and post-injection communication with the study team.

### 2.5 Secondary exploratory outcome measures

To determine changes in headache burden, we collected daily headache intensity NPRS scores (average and maximum) at 1 week, 1 month, and 3 months post-injection. Participants were asked “What was the worst level of headache-related pain you experienced today?” and “During these hours, what was your average level of headache-related pain?” on a scale of 1–10 (10 being the highest) for each day a headache was reported. The maximum and average intensity ratings were averaged for each study timepoint (2 weeks pre-injection and 1 week, 1 month, and 3 months post-injection). Additional exploratory outcomes included headache frequency (number of days headache reported divided to give a weekly average), HIT-6 and QOLIBRI scores at 1 week, 1 month, and 3 months post-injection.

Minimal clinically important difference (MCID) for the NPRS was set as a change of 2 points (33). The MCID for the HIT-6 was taken as a reduction in score of 6 or more points (34) and the QOLIBRI was set as an improvement of at least 30% (30).

### 2.6 Blinding, randomization, and blood collection

Participants were randomized in a 1:1:1 fashion using sealed envelopes prepared by an uninvolved researcher blinded to the study protocol to one of three treatment arms: (1) autologous PRP injection, (2) steroid/anesthetic injection, or (3) normal saline.

All participants underwent a blood draw on their day of injection according to standard phlebotomy technique. Briefly, a 19-gauge needle was used to collect 60 mL of whole blood from the medial vein of the antecubital fossa to a syringe containing 5 mL of Sodium Citrate to prevent coagulation. Whole blood collected from participants in the steroid/anesthetic or saline groups were discarded appropriately. Two syringes (2 mL injectant in each) were prepared by a research assistant and covered to blind the participant and the physician delivering the injection. Following the injection, the physician and the participant were asked to guess which injection they received.

#### 2.6.1 Platelet rich plasma

PRP injections were prepared using the Angel (Arthrex) system, a fully automated PRP preparation machine. The 60 mL of autologous blood was processed via centrifugation (2% hematocrit, spin one 3,500 RPM for 2.56 min, spin two 3,000 RPM for 8.32 min) as per manufacturer instructions (full Arthrex Angel^™^ System processing procedure demonstration available in educational resources on https://www.arthrex.com). This produced 2–3 mL of PRP which was combined with 1–2 mL of platelet poor plasma and divided into two 2 mL syringes.

#### 2.6.2 Steroid/anesthetic

Steroid injections included 20 mg Depo-Medrol mixed with 1.5 mL of 2% lidocaine in each syringe.

#### 2.6.3 Normal saline

Saline injections were prepared using 2 mL of isotonic 0.9% saline in each syringe.

### 2.7 Treatment protocol

On injection day, participants received 2 mL of injectate (PRP, steroid/anesthetic, or normal saline) per side. To reduce pain at the injection site and improve blinding, topical lidocaine cream (5%) was applied approximately 15 min prior to injection. All participants received bilateral GON injections under ultrasound guidance along the superior nuchal line, given the reported advantages over conventional blind injection technique (35). Participants reported their pain using NPRS immediately before and after injection and were monitored for 30 min for immediate adverse reactions.

### 2.8 Data analysis

Recruitment, adverse events, and adherence data are reported descriptively. Headache diary adherence was rated as full (≥99% completed), partial (70–98% completed) or incomplete (≤69% completed) adherence.

Statistical analyses were performed using STATA (v 16.0) software. Demographic characteristics were presented using descriptive statistics (frequencies for count data, means and standard deviations for continuous data), one-way ANOVAs compared group means for continuous data and chi-square tests were performed for categorical data. Exploratory analyses employed multivariable linear regression to investigate mean differences between intake and post-injection follow-up scores in headache average, headache frequency, headache maximum, HIT-6, and QOLIBRI at each follow-up timepoint (1 week, 1 month, and 3 months). An injection type by timepoint interaction term was forced into all models to examine injection type specific score differences at each timepoint. Wald tests assessed the significance of the interaction term for each model but were retained regardless of significance to explore study outcomes stratified by injection type. Estimates and 95% confidence intervals are reported where appropriate. An alpha level of 0.05 was used to determine statistical significance.

## 3 Results

### 3.1 Recruitment

**Figure 1:**
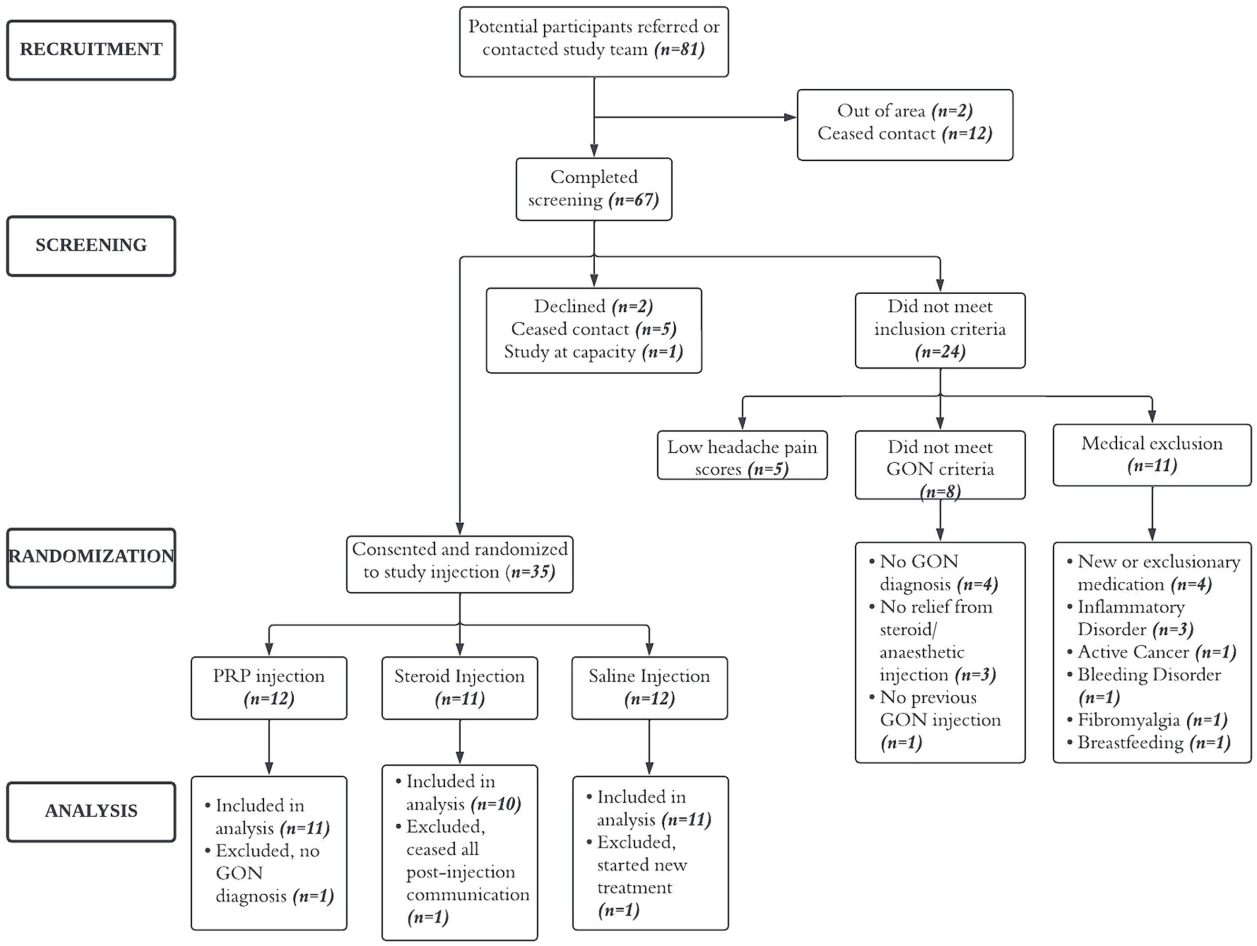
CONSORT diagram. Flow of participants through recruitment and trial.

Between June 2019 and December 2022 (recruitment was on hold from March 2020 to August 2020 due to COVID-19 restrictions), 81 potential participants were referred by physician or contacted the study team, 67 completed the screening phone call and 37 were considered eligible. Of these, two declined to participate, 35 consented and were enrolled into the study. Three participants, one from each group, were excluded from analysis (see Figure 1).

### 3.2 Sample characteristics

Characteristics of the 32 participants included in the analysis are presented in Table 1. The average age was 39.3 years (±11.1) and the majority were female (72%). The most common cause of injury was motor vehicle collision (50%) and participants were recruited on average 46.4 months (range 8–429 months) post-concussion. Of the 32 participants, 13 (41%) had suffered at least 1 previous mTBI and the majority (56%) had received 2–5 therapeutic GON injections. The most common past-medical conditions were previous fractures (20%), surgical interventions (20%), and depression (19%). Past medical history of cancer was significantly higher in the saline group (*χ*^2^ (2) = 7.02, *p* = 0.030), otherwise there were no between group differences (Supplementary Table S1).

**Table 1:** Demographic and clinical characteristics.

	Overall (*n* = 32)	PRP (*n* = 11)	Steroid (*n* = 10)	Saline (*n* = 11)	*p* value
Age in years (mean ± SD)	39.3 ± 11.1	38.3 ± 11.0	42.1 ± 11.6	37.9 ± 11.3	0.650
Sex (female *n* (%))	23 (72%)	8 (73%)	5 (50%)	10 (91%)	0.102
Employment Status					0.058
Student	3 (9%)	2 (18%)	1 (10%)		
Currently working (full or part time)	18 (56%)	7 (64%)	3 (30%)	8 (73%)	
Not currently working but did prior to mTBI	8 (25%)	2 (18%)	3 (30%)	3 (27%)	
Not currently working, did not work prior to mTBI	3 (9%)		3 (30%)		
Injury Mechanism (*n* (%))					0.217
MVC	16 (50%)	6 (55%)	4 (40%)	6 (55%)	
Fall	5 (16%)		3 (30%)	2 (18%)	
Sport/Recreation	10 (31%)	5 (45%)	3 (30%)	2 (18%)	
Work-Related	1 (3%)			1 (9%)	
Time Since Injury in months (mean ± SD)	46.4 ± 72.8	77.0 ± 118.2	32.2 ± 26.6	28.8 ± 15.2	0.232
Previous mTBI (*n* (%))	13 (41%)	2 (18%)	4 (40%)	7 (64%)	0.086
Number of previous GON injections (*n* (%))					0.846
1 Injection	9 (28%)	3 (27%)	4 (40%)	2 (18%)	
2–5 injections	18 (56%)	6 (55%)	5 (50%)	7 (64%)	
6 + injections	5 (16%)	2 (18%)	1 (10%)	2 (18%)	

Demographic data analyzed using ANOVA for continuous variables and chi-square for dichotomous variables. SD, standard deviation; mTBI, mild traumatic brain injury; MVC, motor vehicle collision; GON, greater occipital neuralgia.

### 3.3 Study feasibility

**Figure 2:**
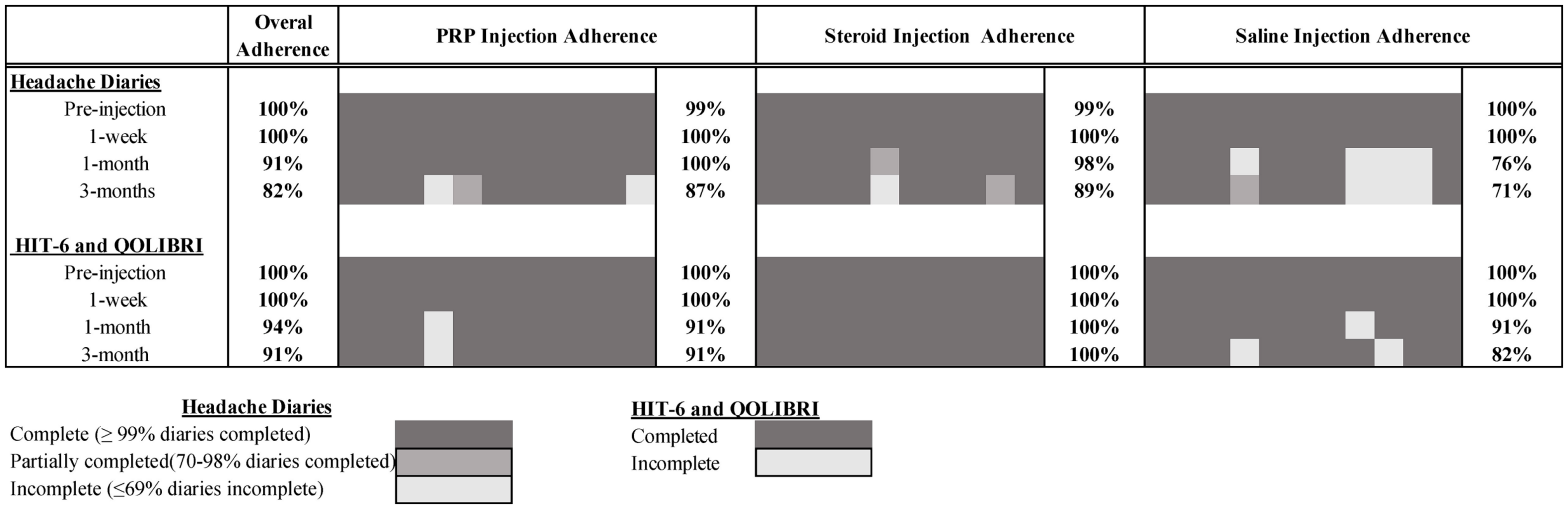
Adherence to outcome measures.

Of the 67 individuals who completed screening calls, 37 (55%) were considered eligible, exceeding our recruitment target of >30%. Of these, 35 (95%) consented and attended the study injection appointment, exceeding our attendance target (>70%). Of the 3 months of post-injection daily headache diaries, 82% were completed (exceeding the target of >70%). Overall retention at 3 months was also >70% for the HIT-6 and QOLIBRI questionnaires (91% completed). Full study adherence by timepoint and injection group is presented in Figure 2.

### 3.4 Safety

No serious adverse events related to any study injection were reported. General post-injection site pain was reported by one participant in the PRP group, two in the saline and two in the steroid/anesthetic group. Participants were advised to use cold pressure and analgesia (no NSAIDS for the first two weeks post-injection) as needed and monitored through communication with the study team and headache diaries.

### 3.5 Integrity of blinding

Less than 50% of participants guessed the correct injection (PRP *n* = 5/11; steroid/anesthetic *n* = 1/10; and saline *n* = 4/11). Half (50%) of participants guessed that they had received the PRP injection with 41% guessing saline and only 9% guessing steroid/anesthetic. Physicians guessed correctly on 2/11 and 3/10 injections in the PRP and steroid/anesthetic group, respectively, and 11/11 following the saline injection. Although physicians were 100% correct following the saline injection, they guessed that the patient had received saline following 75% of the study injections.

### 3.6 Secondary exploratory outcomes

**Figure 3:**
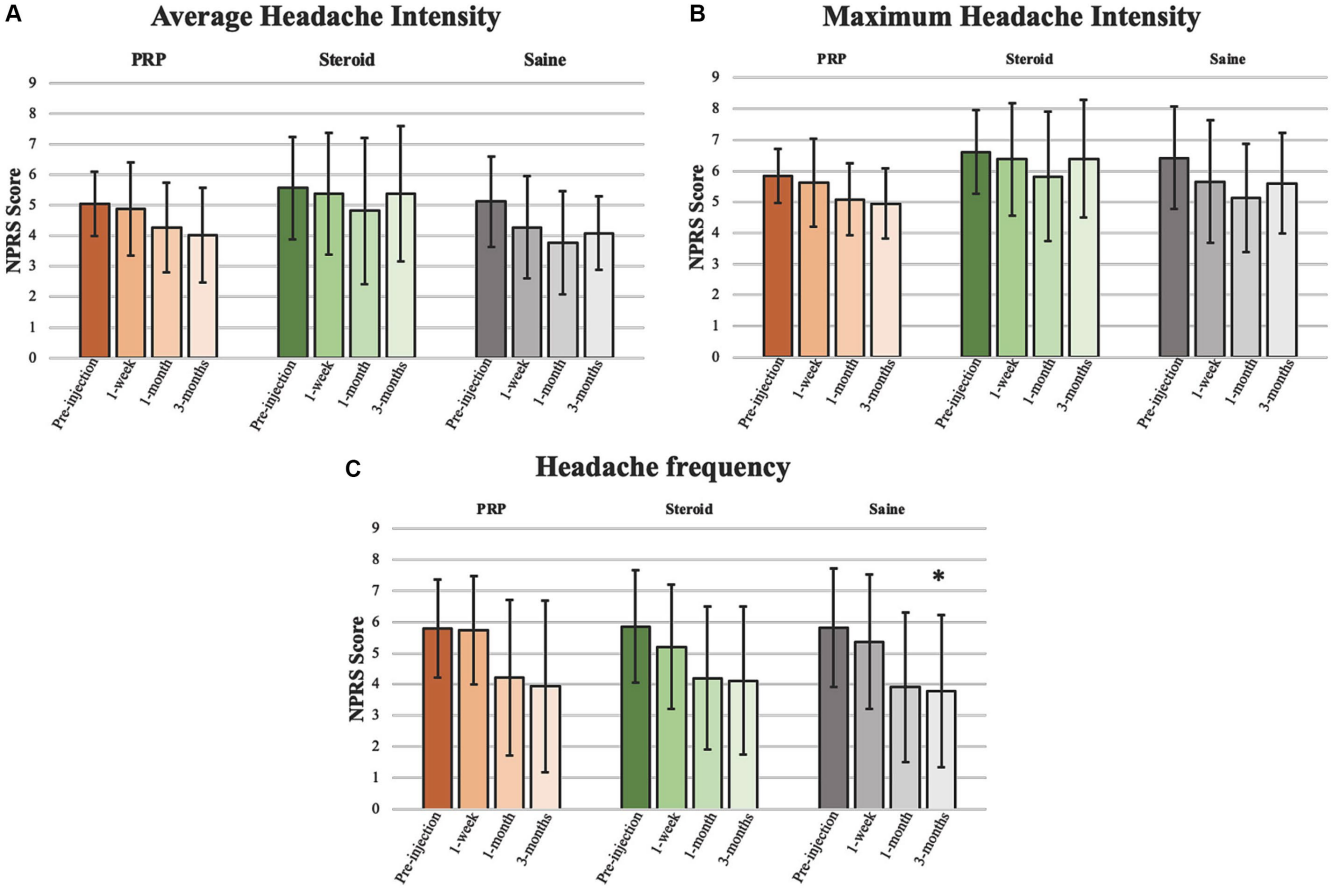
Headache intensity and frequency. Analyses pertaining to **(A)** average headache intensity, **(B)** maximum headache intensity, and **(C)** headache frequency presented as mean ± standard deviation. *Significant at 0.05 level.

**Figure 4:**
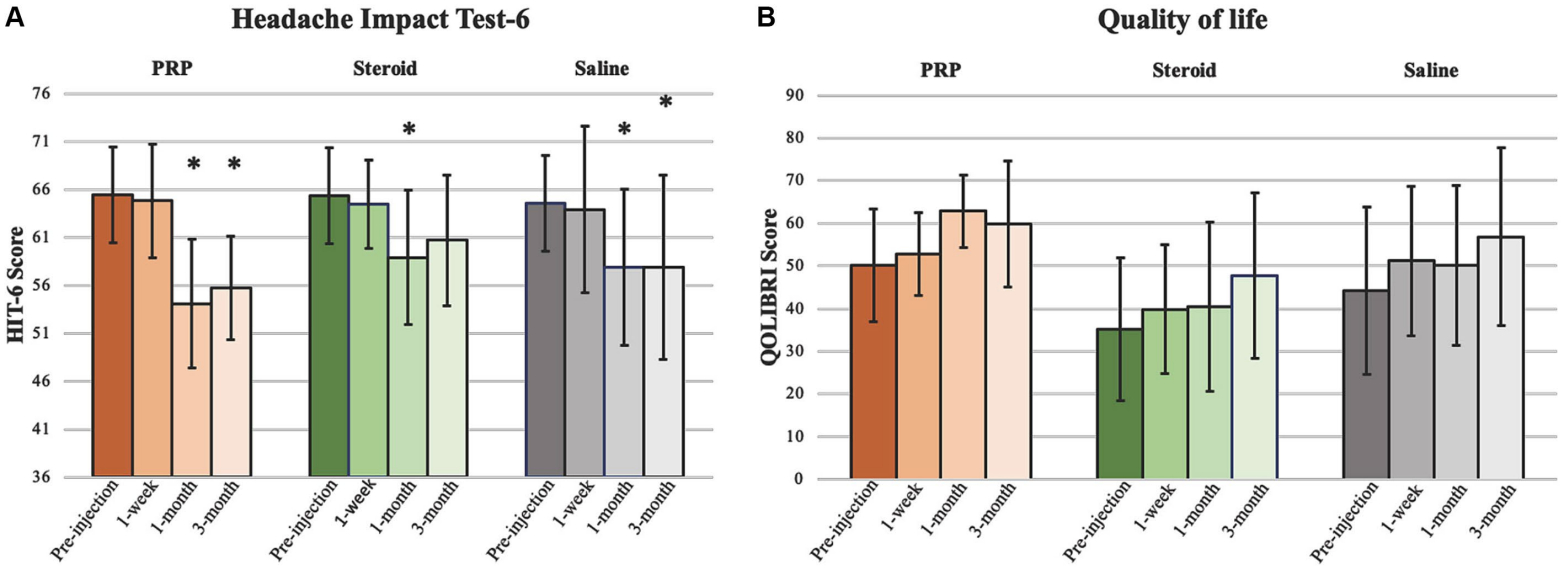
HIT-6 and QOLIBRI. Analyses pertaining to **(A)** Headache Impact Test-6 (HIT-6) and **(B)** Quality of Life after Brain Injury (QOLIBRI) presented as mean ± standard deviation. *Significant at 0.05 level.

Exploratory outcome analyses are presented in Table 2, 3 and Figures 3, 4. There were no significant injection type by timepoint interactions for any of the exploratory outcome measures.

**Table 2:** Exploratory outcome measure scores across study.

	Overall	PRP	Steroid	Saline	Injection by timepoint	
	(*n* = 32)	(*n* = 11)	(*n* = 10)	(*n* = 11)	Wald Test	*p* value
Average headache Severity (mean ± SD)						
Pre-injection	5.2 ± 1.4	5.0 ± 1.0	5.6 ± 1.7	5.1 ± 1.5	0.31	0.933
1-week	4.8 ± 1.7	4.9 ± 1.5	5.4 ± 2.0	4.3 ± 1.7		
1-month	4.3 ± 1.9	4.3 ± 1.5	4.8 ± 2.4	3.8 ± 1.7		
3-months	4.5 ± 1.8	4.0 ± 1.5	5.4 ± 2.2	4.1 ± 1.2		
Maximum headache Severity (mean ± SD)						
Pre-injection	6.3 ± 1.3	5.8 ± 0.9	6.6 ± 1.4	6.4 ± 1.7	0.23	0.965
1-week	5.9 ± 1.7	5.6 ± 1.4	6.4 ± 1.8	5.7 ± 2.0		
1-month	5.4 ± 1.7	5.1 ± 1.2	5.8 ± 2.1	5.1 ± 1.7		
3-months	5.6 ± 1.6	5.0 ± 1.1	6.4 ± 1.9	5.6 ± 1.6		
Headache Frequency (mean ± SD)						
Pre-injection	5.8 ± 1.7	5.8 ± 1.6	5.8 ± 1.8	5.8 ± 1.9	0.06	0.999
1-week	5.4 ± 1.9	5.7 ± 1.7	5.2 ± 2.0	5.4 ± 2.2		
1-month	4.1 ± 2.3	4.2 ± 2.5	4.2 ± 2.3	3.9 ± 2.4		
3-months	3.8 ± 2.4	3.9 ± 2.8	4.1 ± 2.4	3.8 ± 2.4		
QOLIBRI (mean ± SD)						
Pre-injection	43.4 ± 17.3	50.2 ± 13.2	35.2 ± 16.7	44.1 ± 19.6	0.29	0.939
1-week	48.2 ± 15.1	52.8 ± 9.7	39.8 ± 15.1	51.2 ± 17.5		
1-month	51.2 ± 18.4	62.9 ± 8.5	40.5 ± 19.8	50.2 ± 18.7		
3-months	54.7 ± 18.5	59.9 ± 14.8	47.8 ± 19.4	56.8 ± 20.8		
HIT-6 (mean ± SD)						
Pre-injection	65.1 ± 5.8	65.4 ± 5.2	65.3 ± 6.0	64.5 ± 6.8	0.56	0.759
1-week	64.4 ± 6.5	64.8 ± 5.9	64.4 ± 4.6	63.9 ± 8.7		
1-month	57.0 ± 7.3	54.1 ± 6.7	58.9 ± 7.0	57.9 ± 8.1		
3-months	58.1 ± 7.4	55.7 ± 5.4	60.7 ± 6.8	57.9 ± 9.6		

SD, standard deviation; HIT-6, Headache Impact Test-6; QOLIBRI, Quality of Life after Brain Injury.

#### 3.6.1 Headache intensity

Headache intensity was defined as a change in headache severity (average and maximum). There were no significant improvements from pre-injection scores for any injection groups or between groups at any timepoint. Neither average nor maximum headache severity were reduced by a MCID of 2 on the NPRS at any timepoint.

#### 3.6.2 Headache frequency

Headache frequency was determined by the average number of headaches per week. There was a significant improvement in headache frequency from pre-injection following Saline injection at 3 months (*β* = −2.04, 95%CI [−4.109, −0.055], *p* = 0.044). Otherwise, there were no significant changes within PRP or steroid/anesthetic injection groups nor any differences between groups at any timepoint.

#### 3.6.3 QOLIBRI and Hit-6

Analyses pertaining to QOLIBRI and HIT-6 are presented in Figure 4. There was a significant decrease in HIT-6 score from baseline in the PRP (*β* = −11.3, 95%CI [−17.2, −5.3], *p* < 0.001), steroid/anesthetic (*β* = −6.4, 95%CI [−12.5, −0.340], *p* = 0.039) and Saline (*β* = −6.6, 95%CI [−12.6, −0.73], *p* = 0.028) groups at 1 month. This significant decrease was maintained at 3 months in the PRP (*β* −9.7, 95%CI [−15.6, −3.74], *p* = 0.002) and saline group (*β* = −6.7, 95%CI [−12.7, −0.57], *p* = 0.033) but not in the steroid/anesthetic group (*β* = −4.6, 95%CI [−10.7, 1.46], *p* = 0.135). There were no between group differences in score change from baseline.

The MCID for the HIT-6 is a reduction of 6 or more points (34). All groups demonstrated a 6-point decrease in mean scores meeting the MCID at 1 month (Table 2). This change was maintained in the PRP and saline groups but not the steroid/anesthetic group at 3 months. At baseline and at 1 week post-injection, all 3-groups average scores were within the severe impact range for the HIT-6 (≥60 points). At 1 month, the average PRP score was within the ‘some impact’ category (50–55 points) whereas the saline and steroid/anesthetic groups were in the ‘substantial impact’ category (56–59 points). By 3 months, the steroid/anesthetic group had returned to the severe category (mean score of 60.7) whereas the PRP and saline group remained in the ‘substantial impact’ category (scores of 55.7 and 57.9 respectively). The HIT-6 impact grades are presented in Table 4.

**Table 3:** Exploratory outcome analysis: change in score from baseline.

	1-week		1-month		3-months	
	*β* Coef [95% CI]	*p*	*β* Coef [95% CI]	*p*	*β* Coef [95% CI]	*p*
Headache Average						
PRP	−0.049 [−1.52, 1.42]	0.948	−0.65 [−2.12, 0.82]	0.384	−1.04 [−2.59, 0.51]	0.186
Steroid	−0.182 [−1.69, 1.32]	0.811	−0.75 [−2.25, 0.76]	0.329	−0.192 [−1.74, 1.36]	0.806
Saline	−0.85 [−2.28, 0.59]	0.246	−1.35 [−2.98, 0.277]	0.103	−1.04 [−2.61, 0.53]	0.191
Headache Maximum						
PRP	−0.214 [−1.56, 1.13]	0.753	−0.76 [−2.11, 0.59]	0.265	−0.99 [−2.40, 0.43]	0.171
Steroid	−0.23 [−1.64, 1.18]	0.749	−0.79 [−2.20, 0.62]	0.272	−0.210 [−1.66, 1.24]	0.775
Saline	−0.76 [−2.10, 0.59]	0.267	−1.29 [−2.82, 0.23]	0.096	−0.81 [−2.28, 0.65]	0.275
Headache Frequency						
PRP	−0.045 [−1.86, 1.77]	0.961	−1.56 [−3.38, 0.258]	0.092	−1.51 [−3.43, 0.41]	0.122
Steroid	−0.65 [−2.56, 1.26]	0.501	−1.65 [−3.56, 0.257]	0.089	−1.74 [−3.70, 0.221]	0.081
Saline	−0.46 [−2.27, 1.36]	0.621	−1.91 [−3.98, 0.149]	0.069	−2.04 [−4.0, −0.055]	0.044*
QOLIBRI						
PRP	2.58 [−11.4, 16.5]	0.715	12.7 [−1.64, 27.0]	0.082	9.6 [−4.7, 23.9]	0.185
Steroid	4.6 [−10.0, 19.2]	0.535	5.3 [−9.3, 20.0]	0.471	12.6 [−2.07, 27.2]	0.092
Saline	7.2 [−6.8, 21.1]	0.311	6.1 [−8.2, 20.4]	0.396	12.7 [−2.01, 27.4]	0.090’
HIT-6						
PRP	−0.55 [−6.3, 5.2]	0.852	−11.3 [−17.3, −5.3]	<0.001*	−9.7 [−15.6, −3.74]	0.002*
Steroid	−0.90 [−7.0, 5.2]	0.769	−6.4 [−12.5, −0.340]	0.039*	−4.6 [−10.7, 1.46]	0.135
Saline	−0.64 [−6.4, 5.1]	0.828	−6.6 [−12.6, −0.73]	0.028*	−6.7 [−12.7, −0.57]	0.033*

* indicates significant difference from baseline at 0.05 level. HIT-6, Headache Impact Test-6; QOLIBRI, Quality of Life after Brain Injury.

**Table 4:** Impact grade of the HIT-6.

	Little or none *n* (%)	Moderate *n* (%)	Substantial *n* (%)	Severe *n* (%)
Pre-Injection				
PRP	0 (0%)	1 (9%)	1 (9%)	9 (82%)
Steroid	0 (0%)	0 (0%)	2 (20%)	8 (80%)
Saline	0 (0%)	1 (9%)	1 (9%)	9 (82%)
1-week				
PRP	0 (0%)	0 (0%)	3 (27%)	8(73%)
Steroid	0 (0%)	0 (0%)	1 (10%)	9 (90%)
Saline	1 (9%)	1 (9%)	2 (18%)	7 (64%)
1-month				
PRP	3 (30%)	5(50%)	0 (0%)	2 (20%)
Steroid	2 (20%)	1(10%)	2 (20%)	5 (50%)
Saline	1(10%)	3 (30%)	2 (20%)	4 (40%)
3-months				
PRP	1 (10%)	4 (40%)	3 (30%)	2 (20%)
Steroid	0 (0%)	3 (30%)	1 (10%)	6 (60%)
Saline	1 (11%)	3 (33%)	1 (11%)	4 (44%)

Little or none = HIT-6 score of <49, Moderate = score of 50–55, Substantial = score of 56–59, Severe = score of 60–78. Displayed as number of participants (%). HIT-6, Headache Impact Test-6.

There were no significant increases in QOLIBRI score from baseline in any of the injection groups or between group comparisons at any timepoint. The MCID for the QOLIBRI is estimated to be a 30% increase in overall score (30). This was only achieved in the steroid/anesthetic group at 3 months post-injection (35.7% increase in group score).

## 4 Discussion

To our knowledge, this is the first prospective, randomized, controlled, double-blinded clinical trial demonstrating the feasibility and safety of a single PRP injection for treating post-traumatic GON following a concussion. First, we were successful in meeting our primary recruitment targets, with 86% of recruited patients included in the final analysis. With regards to adherence and retention, over 80% of the daily headache diaries were completed over 3 months and over 90% completed HIT-6 and QOLIBRI questionnaires. Second, there were no serious adverse events or reactions reported in any group relating to the intervention. Exploratory outcomes revealed there were no significant differences between groups for headache intensity, frequency, headache functional outcomes (HIT-6) or quality of life (QOLIBRI) at any time point. Thus, the clinical effectiveness of PRP was comparable to saline and steroid/anesthetic injections but larger powered studies are necessary to evaluate efficacy.

Although less studied, PRP has recently emerged as a potential treatment in peripheral nerve disorders. Perineural PRP has been shown to improve pain and function in carpal tunnel syndrome (24–27) and diabetic polyneuropathy (23) with studies demonstrating superior duration of effect for PRP for as long as one year in some cases (25, 26). The mechanisms of pain in post-traumatic GON are believed to be similar to other peripheral nerve disorders, where entrapment, irritation, and damage occurs along the course of the nerve (12, 13, 16). Nerve compression can lead to intraneural ischemia and edema, which can result in chronic neurogenic inflammation and pain (36). The exact mechanisms through which PRP improves pain and nerve function are unknown, but it is thought to reduce local inflammation, stimulate tissue repair and encourage angiogenesis (22). Animal studies have demonstrated PRP’s ability to assist in remyelination and axonal regeneration (37–40), while studies in humans have demonstrated improved electroconductivity following PRP injection (23, 25, 27). These findings highlight PRP’s potential to restore nerve function and augment the neural microenvironment, which may account for its prolonged duration of effect. Whereas conventional treatment with cortisone can reduce pain transiently via anti-inflammatory effect, lasting on average 4 weeks in post-traumatic GON (13), it may hinder long term tissue repair. Furthermore, repeated steroid injections can cause adverse local and systemic effects, making them a less desirable treatment over the long term.

Another possible mechanism of action for perineural injections is the biomechanical effect of fluid itself being injected, termed ‘hydrodissection’. The volume of injectate, regardless of the substance, can help loosen any scaring or adhesions around the nerve causing entrapment, thereby improving blood flow and nerve mobility. There is some evidence that hydrodissection with saline alone can improve peripheral nerve pain (41), which may in part, account for the similar effects observed across our study groups, regardless of type of injection received. More research is needed to establish the exact mechanisms by which perineural injections exert their effect.

Despite increased interest in PRP, there remains significant methodological heterogeneity with regards to the preparation and composition. This includes devices used in preparation (commercial kits/local equipment, centrifugation, volume of whole blood), dosage, concentration of platelets, choice of anticoagulant (sodium citrate, ACD-A etc.), and the presence or absence of leukocytes and fibrin (19, 26). These variations in methodology contribute to the diverse outcomes seen in the PRP literature. Standardization of PRP protocols, particularly for nerve injury will be helpful to compare study outcomes. Our study used a similar approach to other peripheral nerve/PRP studies suggesting this study could be replicated with a larger sample size.

The findings from this study echo those from other clinical trials, which have established the efficacy and safety of PRP, reporting few or no adverse events following injection (17, 23, 25). When compared to corticosteroids, randomized control trials and meta-analysis have found that although corticosteroids may be more beneficial in the short term, pain scores at longer follow-ups found PRP to be equal, if not better with regard to pain improvement in lateral epicondylitis (20), rotator cuff tears (18), and plantar fasciitis (42). This is reflective in our results with reductions in the total HIT-6 and HIT-6 pain severity score maintained at the 3 month point in the PRP group and saline groups, but not steroid/antiesthetic group.

### 4.1 Limitations

Exploratory outcomes evaluated the effectiveness of a PRP injection compared to a steroid/anesthetic or saline injection. A full evaluation of the benefits of PRP over current treatment practices for post-traumatic GON requires a larger sample size. As the primary objective of this pilot study was to determine feasibility and safety of the protocol prior to larger trials, we did not complete a power calculation but assumed that a sample size of 30 would be sufficient for our aims and objectives (16, 43). Although the treatment groups were similar in baseline demographics, the majority of participants were female, which has been shown to be independent risk factor for poor symptomatic recovery following TBI (3) and may have affected therapeutic response. As well, we did not perform platelet counting for PRP samples, but we assume that concentrations were adequate with the injections being prepared according to manufacturer’s specifications. Further, persistent benefits from PRP injection beyond 3 months was not assessed but would be important to evaluate in future studies. Lastly, while we have demonstrated the feasibility of our study protocol for future study design, we did not assess the clinical or economic feasibility of PRP injections under ultrasound guidance. When compared to steroid/anesthetic and saline injections, PRP requires more equipment and is more expensive to administer, which may limit its clinical application.

## 5 Conclusion

This novel study demonstrates the feasibility and safety of a single PRP injection in treating post-traumatic GON following concussion. Preliminary exploratory outcomes suggest that a single PRP injection was comparable to steroid/anesthetic and saline. Further studies with a larger sample size and longer duration of follow-up are indicated.

## Data availability statement

The datasets presented in this article are not readily available but data may be made available on completion of a data sharing agreement with the corresponding author and the University of Calgary. Requests to access the datasets should be directed to cdebert@ucalgary.ca.

## Ethics statement

The studies involving humans were approved by Conjoint Health Research Ethics Board, University of Calgary. The studies were conducted in accordance with the local legislation and institutional requirements. The participants provided their written informed consent to participate in this study.

## Author contributions

JS: Writing – original draft, Writing – review & editing, Conceptualization, Methodology, Supervision, Visualization, Funding acquisition. CC: Writing – original draft, Writing – review & editing, Data curation, Project administration. JT: Writing – original draft, Writing – review & editing, Data curation, Formal analysis. SB: Writing – original draft, Writing – review & editing, Data curation, Formal analysis. MM: Writing – original draft, Writing – review & editing, Data curation, Project administration. RS: Conceptualization, Funding acquisition, Methodology, Supervision, Writing – original draft, Writing – review & editing, Resources, Visualization. CD: Conceptualization, Writing – original draft, Writing – review & editing, Funding acquisition, Methodology, Resources, Supervision, Visualization.
