# Supplementary material for: Ultrasound guided platelet rich plasma injections for post-traumatic greater occipital neuralgia following concussion: a pilot randomized controlled trial
Source: Front Neurol. 2024 Jun 7;15:1400057. doi: 10.3389/fneur.2024.1400057 (PMC11191875; doi:10.3389/fneur.2024.1400057)
Supplement: Supplementary file 1 [file Table_1.DOCX]

Supplementary material

**Supplementary table 1.**

|  | **Overall** | | **PRP Injection** | **Steroid Injection** | **Saline Injection** |  |
| --- | --- | --- | --- | --- | --- | --- |
|  | | **(n=32)** | **(n=11)** | **(n=10)** | **(n=11)** |  |
|  | |  |  |  |  |  |
| **Height in cm (mean ± SD)** | | 166.5 ± 11.3 | 168.0 ± 10.3 | 168.7 ± 16.9 | 163.2 ± 5.4 | F(2,28) = 0.71, *p*=0.499 |
| **Weight in kg (mean ± SD)** | | 82.5 ± 20.9 | 88.3 ± 26.6 | 79.5 ± 19.7 | 79.1 ± 15.2 | F(2,28) = 0.65, *p*=0.530 |
|  | |  |  |  |  |  |
| **Past Medical History (n(%))** | |  |  |  |  |  |
| Diabetes (Type II) | | 1 (3%) | 1 (9%) |  |  | χ2(2) = 2.20, *p*=0.333 |
| Hypertension | | 5 (16%) | 2 (18%) | 2 (10%) | 1 (11%) | χ2(2) = 0.60, *p*=0.742 |
| Sleep Apnea | | 5 (16%) | 2 (18%) | 1 (10%) | 2 (18%) | χ2(2) = 0.37, *p*=0.830 |
| Sleep Disorder | | 5 (16%) | 2 (18%) |  | 3 (27%) | χ2(2) = 4.42, *p*=0.110 |
| Asthma | | 9 (28%) | 1 (9%) | 3 (30%) | 5 (45%) | χ2(2) = 3.95, *p*=0.139 |
| Liver Disease | | 3 (9%) | 1 (9%) |  | 2 (18%) | χ2(2) =2.78, *p*=0.249 |
| Vitamin Deficiency | | 4 (13%) | 3 (27%) |  | 1 (11%) | χ2(2) = 4.52, *p*=0.104 |
| Endocrine Disorder | | 1 (3%) | 1 (9%) |  |  | χ2(2) = 2.20, *p*=0.333 |
| Gastrointestinal Disease | | 3 (9%) | 1 (9%) | 1 (10%) | 1 (11%) | χ2(2) = 0.01, *p*=0.997 |
| Ear-Nose-Throat Disorder | | 2 (6%) |  | 1 (10%) | 1 (11%) | χ2(2) = 1.76, *p*=0.415 |
| MSK Disorder | | 3 (9%) |  | 1 (10%) | 2 (18%) | χ2(2) = 2.98, *p*=0.225 |
| Fracture (Broken Bone) | | 20 (63%) | 7 (64%) | 6 (60%) | 7 (64%) | χ2(2) = 0.39, *p*=0.981 |
| Autoimmune disorder | | 1 (3%) |  |  | 1 (11%) | χ2(2) = 2.20, *p*=0.333 |
| Seizure or Epilepsy | | 2 (6%) |  |  | 2 (18%) | χ2(2) = 4.53, *p*=0.104 |
| Cancer or Tumor | | 3 (9%) |  |  | 3 (27%) | χ2(2) = 7.02, *p*=0.030* |
| Depression | | 19 (59%) | 7 (64%) | 6 (60%) | 6 (55%) | χ2(2) = 0.19, *p*=0.909 |
| Anxiety Disorder | | 16 (50%) | 6 (55%) | 5 (50%) | 5 (45%) | χ2(2) = 0.18, *p=*0.913 |
| Learning Disability | | 5 (16%) | 1 (9%) | 1 (10%) | 3 (27%) | χ2(2) = 1.64, *p*=0.440 |
| ADHD/ADD | | 5 (16%) | 3 (27%) | 2 (10%) |  | χ2(2) = 4.84, *p*=0.890 |
| Substance Use Disorder | | 1 (3%) |  | 1 (10%) |  | χ2(2) = 2.40, *p*=0.301 |
| PTSD | | 11 (34%) | 4 (36%) | 3 (30%) | 4 (36%) | χ2(2) = 0.13, *p*=0.939 |
| Personality Disorder | | 1 (3%) | 1 (9%) |  |  | χ2(2) = 2.20, *p*=0.333 |
| Other Psychiatric Disorder | | 1 (3%) | 1 (9%) |  |  | χ2(2) = 2.20, *p*=0.333 |
| Surgical Intervention | | 20 (63%) | 7 (64%) | 5 (50%) | 8 (73%) | χ2(2) = 1.17, *p*=0.558 |
|  | |  |  |  |  |  |

*Note:* Demographic data analyzed using ANOVA for continuous variables and chi-square for dichotomous variables. * Statistically significant at p<0.05. Surgical Intervention referring to surgery other than head and/or neck (exclusionary). *Abbreviations*: SD, standard deviation; MSK, musculoskeletal; ADHD/ADD, attention deficit (hyperactive) disorder; PTSD, post-traumatic stress disorder.
